# β-glucan: a potent adjuvant in immunotherapy for digestive tract tumors

**DOI:** 10.3389/fimmu.2024.1424261

**Published:** 2024-07-19

**Authors:** Meiyu Wang, Jinhua Pan, Wu Xiang, Zilong You, Yue Zhang, Junyu Wang, Anren Zhang

**Affiliations:** ^1^ Department of Rehabilitation Medicine, Shanghai Fourth People’s Hospital Affiliated to Tongji University School of Medicine, Shanghai, China; ^2^ School of Chinese Medicine, Hong Kong Baptist University, Hong Kong, Hong Kong SAR, China; ^3^ Department of Ophthalmology, Chengdu Pidu District Hospital of Traditional Chinese Medicine, Chengdu, China; ^4^ School of Health Preservation and Rehabilitation, Chengdu University of Traditional Chinese Medicine, Chengdu, China; ^5^ Department of Biochemistry and Biophysics, School of Basic Medical Sciences, Peking University, Beijing, China

**Keywords:** beta glucan, β-glucan receptor, mechanism, immunotherapy, digestive tract tumors

## Abstract

The immunotherapy for gastrointestinal tumors, as a significant research direction in the field of oncology treatment in recent years, has garnered extensive attention due to its potential therapeutic efficacy and promising clinical application prospects. Recent advances in immunotherapy notwithstanding, challenges persist, such as side effects, the complexity of the tumor immune microenvironment, variable patient responses, and drug resistance. Consequently, there is a pressing need to explore novel adjunctive therapeutic modalities. β-glucan, an immunomodulatory agent, has exhibited promising anti-tumor efficacy in preclinical studies involving colorectal cancer, pancreatic cancer, and gastric cancer, while also mitigating the adverse reactions associated with chemotherapy and enhancing patients’ quality of life. However, further clinical and fundamental research is warranted to comprehensively evaluate its therapeutic potential and underlying biological mechanisms. In the future, β-glucan holds promise as an adjunctive treatment for gastrointestinal tumors, potentially bringing significant benefits to patients.

## 1 Introduction

In addition to severe gastrointestinal dysfunction, gastrointestinal tumor patients often present with clinical symptoms such as fatigue (24.9%), numbness or tingling sensation (17.2%), abdominal distension (17.2%), dry mouth (15.9%), memory difficulties (11.8%), and severe pain (32%, 47/145), these high symptom burdens may be correlated with a decreased quality of life among gastrointestinal tumor survivors, and the significant economic burden resulting from cancer treatment is also associated with a lower quality of life (1). Currently, the main clinical treatments for gastrointestinal tumors are surgery and radiotherapy/chemotherapy. However, these therapies often result in adverse reactions such as leukopenia, thrombocytopenia, anorexia, anemia, diarrhea, liver damage, mucositis, and dysphagia (2, 3). Tumor immunotherapy is an essential adjuvant therapy following tumor surgery, chemotherapy, and radiotherapy, with the advantages of superior specificity, broad anti-tumor spectrum, and mild toxic side effects (4–6). In recent years, tumor immunotherapy has gradually been applied in the treatment of gastrointestinal tumors, representing a therapeutic approach that activates or enhances the patient’s immune system to attack and eliminate tumor cells (7). Despite significant progress achieved in recent years, it still faces multiple challenges, including: 1) the presence of certain side effects such as diarrhea, fatigue, rash, and elevated lipase levels (8); 2) the complexity of the tumor immune microenvironment, which can affect the effectiveness of immunotherapy (7); 3) the heterogeneity in the response of different patients to specific immunotherapy due to individual differences (9, 10); 4) the development of resistance and low response rates to immune checkpoint inhibitors, resulting from specific genetic mutations or upregulated gene expression in tumor cells or immune cells (7, 11). Therefore, we need to explore novel adjunctive therapies for clinical treatment of gastrointestinal tumors.

β-Glucan is widely used as a natural modulator of biological effects in the study of tumor immunotherapy. In recent years, relevant studies have confirmed that (12), as an immune modulator, β-glucan has shown positive effects in inhibiting tumor cell proliferation and activating anti-tumor immune responses (13–16). Meanwhile, due to its stable structure, it is also used as a component of oral anti-tumor drug delivery carriers (17).

The most common β-glucans used in clinical applications include yeast glucan, shiitake mushroom polysaccharides, fission mushroom polysaccharides, and oat glucan;The source, structure, water solubility and molecular weight of these different β-glucans influence the intensity and type of immune response induced by them (18). β-glucans can be categorized into particulate and soluble glucans based on their water solubility. It has been shown that particulate β-glucan can activate dendritic cells (DCs) and macrophages through the dectin-1 pathway (19, 20). However, soluble glucans can also enhance macrophage proliferation and dectin-1 expression by inducing granulocyte-macrophage colony stimulating factor (GM-CSF) production in macrophages via dectin-1-independent ERK and p38 MAPK pathways, and ultimately exert antitumor effects by mediating TNF-α production via the Syk pathway (21). β-Glucan emerges as a promising biological effector modulator, exhibiting both safety and efficacy in modulating innate and adaptive immune responses (22). It triggers a cascade of immune defense reactions by interacting with surface receptors of diverse immune cells, indicating its potential as a therapeutic agent in immunomodulatory strategies (23).

Gastrointestinal tumors are becoming one of the deadliest diseases worldwide. β-Glucan, a powerful immunomodulator, has exhibited remarkable anti-tumor efficacy in preclinical investigations targeting colorectal, pancreatic, and gastric cancers (14, 24, 25). Notably, its high stability underscores its significance as a carrier for targeted drugs specifically designed to combat gastric cancer (17). Clinical studies further validate its ability to enhance the 5-year survival rate in patients with hepatocellular carcinoma, gastric cancer, and colorectal cancer, while simultaneously reducing the rate of recurrence (26–28). Moreover, β-glucan alleviates adverse reactions associated with chemotherapy, including nausea, abdominal discomfort, mucositis, diarrhea, and leukopenia/thrombocytopenia (29), ultimately leading to an enhancement in patients’ overall quality of life (30). In the future, with the advancement of clinical research and technological innovations, β-glucan holds the promise of emerging as a significant adjunctive therapeutic option for patients with gastrointestinal tumors.

## 2 Classification and sources of β-glucans

β-Glucan, a versatile polysaccharide, exhibits a wide range of biological functions and is found ubiquitously in diverse biological entities, including Cereals, fungi, algae and bacteria, primarily in the forms of (1 → 3) (1 → 6)-β-D-glucan, (1 → 3) (1 → 4)-β-D-glucan and (1 → 3)-β-D-glucan, in addition, studies have shown that the branching derived from the glycosidic chain core varies among different species (**Table 1**). Cereals constitute the primary source of β-glucans. Specifically, wheat typically contains approximately 1% β-glucans, oats range from 3% to 7%, and barley exhibits a β-glucan content spanning from 5% to 11%, indicating their significant presence in these cereal grains (48). Naturally occurring β-glucans derived from cereals exhibit a distinctive structural motif that primarily comprises β-(1→3) and (1→4) glycosidic linkages, which underlie their unique chemical constitution and diverse physicochemical properties. These β-glucans in cereals are linear homopolysaccharides composed of D-glucopyranosyl residues linked through β-(1→3) and β-(1→4) glycosidic bonds (49).In contrast to cereal-derived β-glucans, β-glucans isolated from fungi and bacteria possess a distinct structural composition, primarily characterized by β-(1→3) and (1→6) glycosidic linkages. In these microorganisms, β-glucans consist of a linear backbone composed of glucose residues interconnected via β-(1→3) linkages, often adorned with glucose side-chains of varying lengths, which are attached through β-(1→6) linkages (50). On average, β-(1→6) substitutions occur at intervals of every two to three β-(1→3) main chain residues.

**Table 1:** A summary of the sources of β-glucans.

Source	Organism/species	Glycosidic linkages	Reference
Cereals	*oats*	β-(1,3; 1,4)	(31)
	*barley grain*	β-(1,3; 1,4)	(32)
	*rye*	β-(1,3; 1,4)	(33)
	*wheat*	β-(1,3; 1,4)	(34)
	*rice*	β-(1,3; 1,4)	(33)
	*Hordeum vulgare*	β-(1,3; 1,4)	(35)
	*Avena sativa*	β-(1,3; 1,4)	(36)
Fungi	*Grifola frondosa*	β-(1;3, 1;6)	(37)
	*Lentinula edodes*	β-(1;3, 1;6)	(38)
	*Saccharomyces cerevisiae*	β-(1;3, 1;6)	(39)
	*Trametes versicolor*	β-(1;3, 1;4)	(40)
	*Schizophyllum commune*	β-(1;3, 1;6)	(41)
	*Sclerotium rolfsii*	β-(1;3, 1;6)	(42)
	*Saccharomyces cerevisiae*	β-(1;3, 1;6)	(43)
	*yeast*	β-(1;3, 1;6)	(44)
	*mushroom*	β-(1;3, 1;6)	(45)
Algae	*Laminaria digitata*	β-(1;3, 1;6)	(42)
	*seaweed*	β-(1;3, 1;6)	(46)
	*microalgae*	β-(1;3)	(47)
Bacteria	*Alcaligenes faecali*	β-(1;3)	(35)

## 3 Biosynthetic Pathways of β-glucan

**Figure 1:**
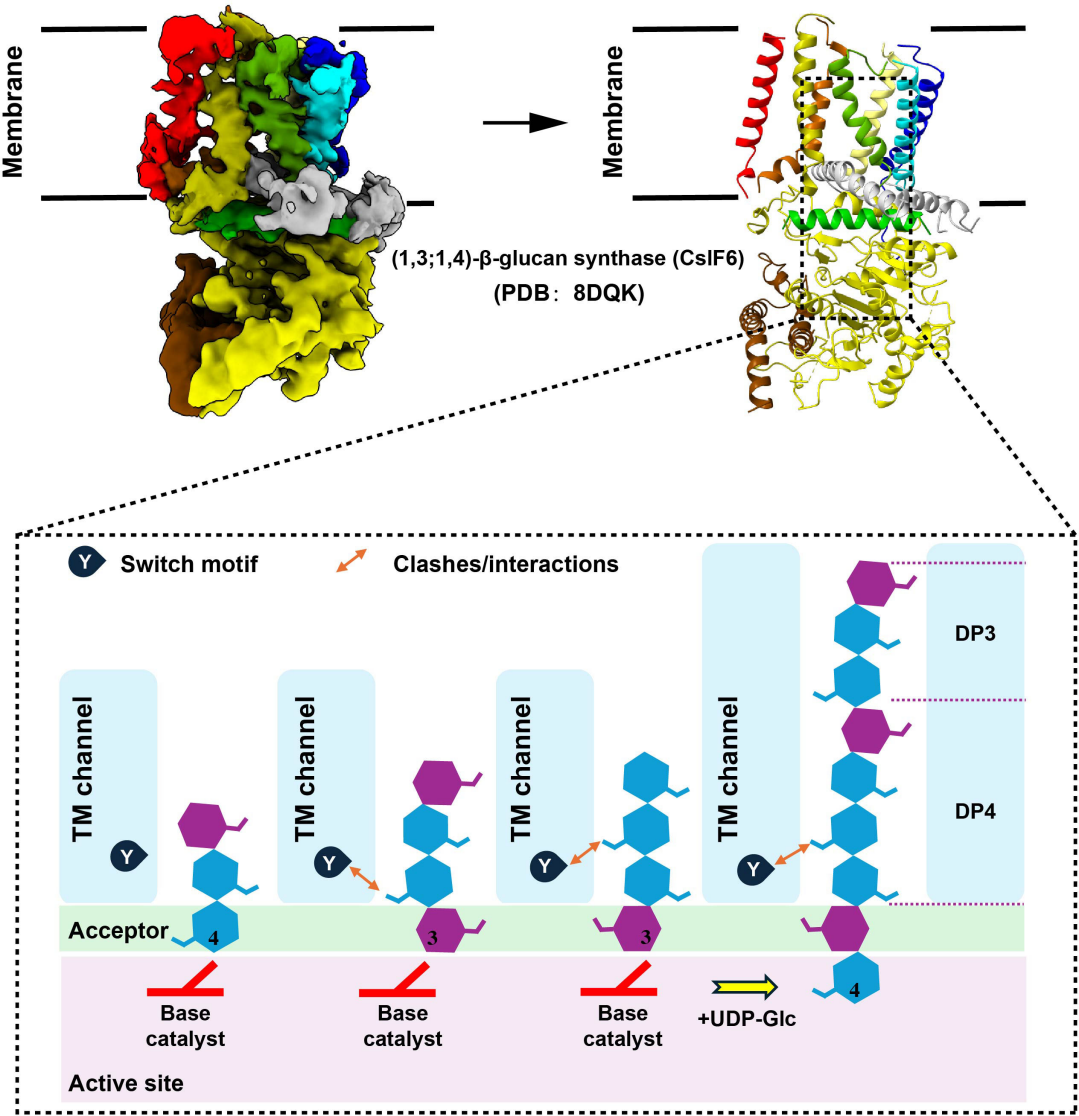
Model of (1, 3; 1, 4)-β-glucan biosynthesis. To generate figure using the raw data from the PDB database (PDB: 8DQK), cartoon representations of the protein structures were created using PyMOL. The graphical approach for illustrating the synthetic mechanism is referenced from DOI: 10.1126/sciadv.add1596.

Recently, Pallinti Purushotham et al. (51) have identified the catalytic activity and protein structural characteristics of barley (1, 3; 1, 4)-β-glucan synthase (CslF6) through cryo-electron microscopy and biochemical experiments, revealing the detailed mechanism of CslF6-catalyzed (1, 3; 1, 4)-β-glucan biosynthesis. Specifically, the C6 hydroxyl groups of consecutive (1, 3)- and (1, 4)-β-linked glycosyl units point in approximately the same direction during translocation, while consecutive (1, 4)-β-linked glycosyl units rotate approximately 180°. A conserved “ switch motif “ at the entrance of the transmembrane (TM) channel serves as a crucial element for CslF6 enzymatic activity. This motif recognizes specific features of the translocating glucan, interacts with the penultimate glucosyl unit, leading to the formation of a new cellotriosyl unit (DP3) (51). The “switch motif” can also interact with the antepenultimate glucosyl unit, triggering the repositioning of the glucan acceptor, ultimately resulting in the formation of a new cellotetraosyl unit (DP4) (51). This interaction with the penultimate and antepenultimate glucosyl units ultimately generates (1, 3; 1, 4)-β-glucan containing DP3 and DP4 (**Figure 1**).

**Figure 2:**
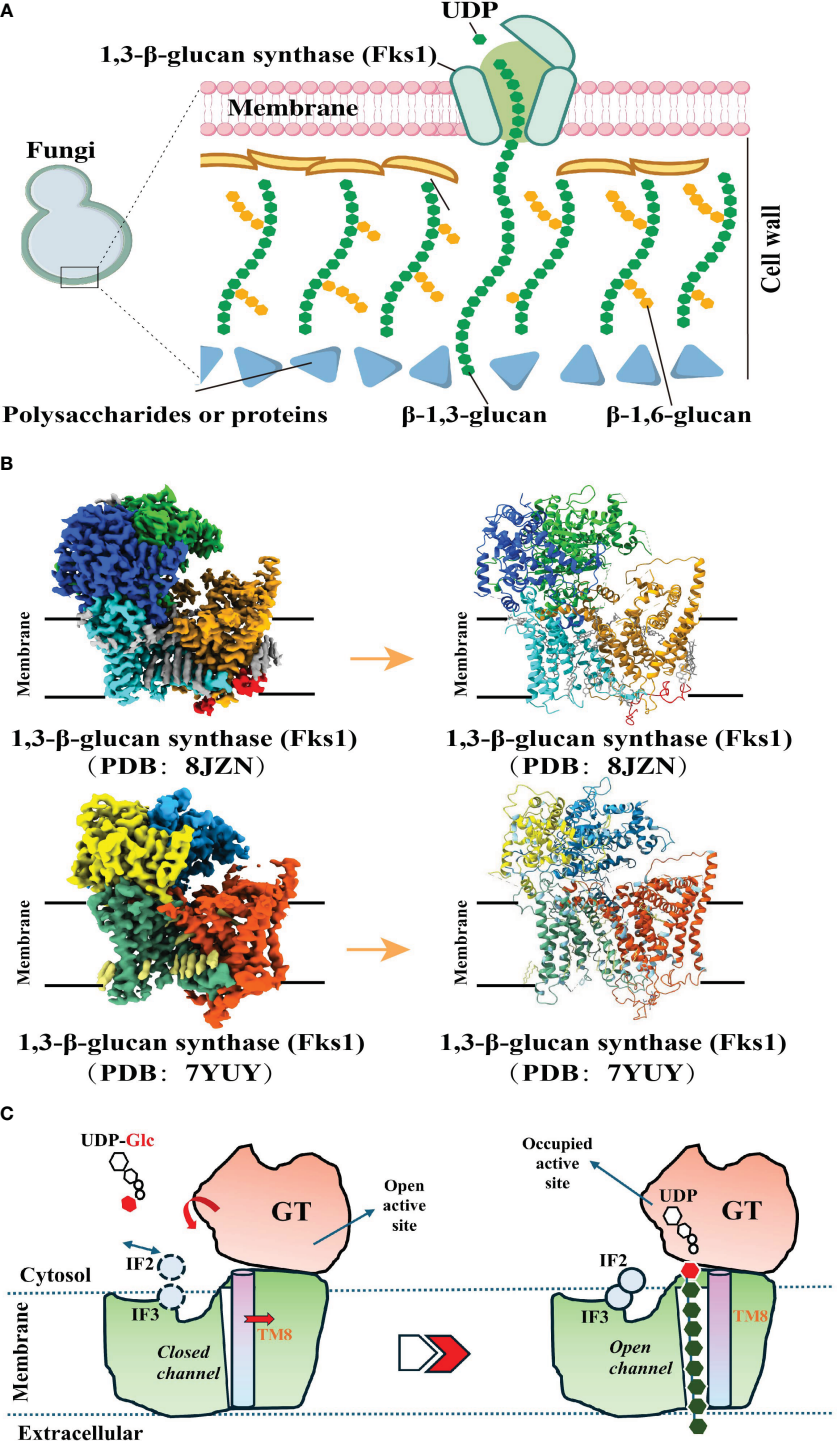
Biosynthetic Pathways of 1, 3-β-Glucan. **(A)**. A model of the fungal cell wall depicts FKS1 utilizing UDP-Glc as a substrate to synthesize the crucial β-1,3-glucan component. **(A)** was created using Figdraw (https://www.figdraw.com. ID : TROPO6a95 d). **(B)**. Cryo-EM map of FKS1 and the FKS1 structure in cartoon display. To generate figures using the raw data from the PDB database (PDB: 8JZN and PDB: 7YUY), cartoon representations of the protein structures were created using PyMOL. **(C)**. Proposed catalytic mechanism of Fks1. The graphical approach for illustrating the synthetic mechanism is referenced from DOI: 10.1126/sciadv.adh7820 and DOI: 10.1038/s41586-023-05856-5.

In the biosynthetic pathway of β-1,3-glucan, the pivotal membrane-integral Glucan Synthetase (GS) complex (**Figure 2A**), comprised of a fks/gsc-encoded catalytic subunit and a rho1-encoded regulatory subunit, holds paramount significance (52). Recent studies have made groundbreaking progress by utilizing cryo-electron microscopy (Cryo-EM) to elucidate the structure of fungal 1,3-β-glucan synthase (Fks1) (53, 54) (**Figure 2B**). This significant advancement has profoundly contributed to our understanding of the β-1,3-glucan synthesis mechanism. Specifically, the Fks1 catalytic subunit necessitates the soluble GTPase Rho1 as its regulatory partner, enabling uridine diphosphate glucose (UDP-Glc) to serve as the sugar donor for the catalysis of β(1→3) glycosidic bonds in 1,3-β-glucan, thereby promoting the translocation of glucan chains from the intracellular milieu to the extracellular space (52, 53). The cryo-EM structure reveals the apo state, characterized by the flexibility of IF2 and IF3 and an unoccupied active site primed for substrate binding. In this state, the glucan transport channel remains closed, held in place by TM8. However, upon substrate binding and the subsequent chemical reaction, the GT domain undergoes a downward motion, expelling the formed glucan product out of the cell. Simultaneously, IF2 and IF3 play a role in stabilizing the glucan chain during its elongation. Notably, the movement of TM8 shifts outward, opening the channel for glucan release (53). (**Figure 2C**).

The research indicates that CSLD3 is a UDP-glucose-dependent β-1,4-glucan synthase (55). However, due to the lack of structural elucidation, the specific biosynthetic pathway involving this protein remains unreported. Although KRE6, SKN1, FfGS6, and GsmA have been proven to participate in β-1,6-glucan synthesis, the qualitative characterization of the activities of these β-1,6-glucan synthases has not been achieved, and their protein structures have yet to be successfully resolved. Consequently, the regulatory mechanisms underlying their function remain unclear (56–58).

## 4 Receptors that mediate the effects of β-glucan

**Figure 3:**
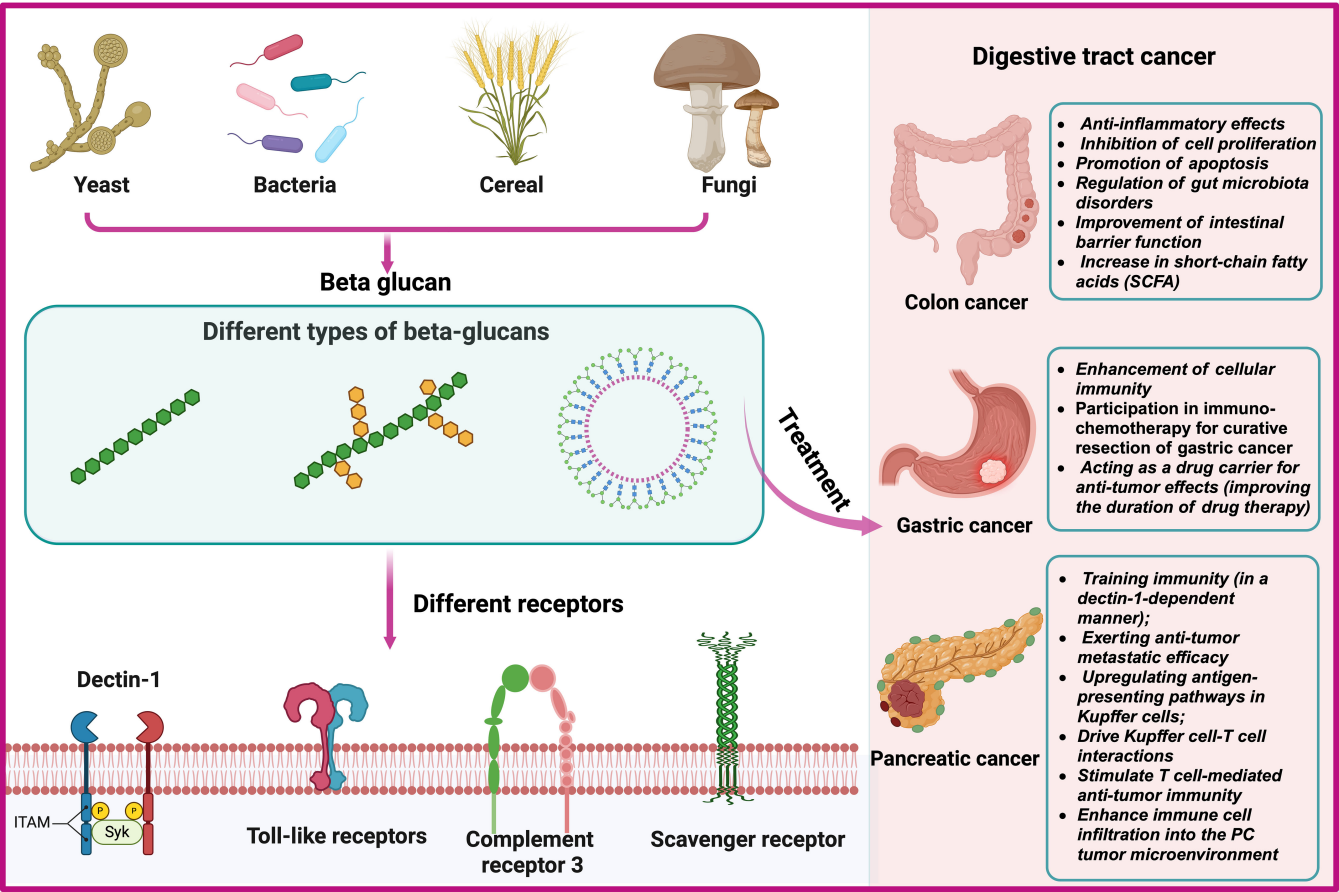
Sources, Classification and Interacting Receptors of Beta-Glucans and Biological Mechanisms for the Prevention and Treatment of Gastrointestinal Tumors.

The immune regulatory function of β-glucan depends on the complexity of their conformational changes (59). β-glucan can regulate innate immune cells by binding to pattern recognition receptors (PRR) on the surface, thereby exerting anti-tumor effects (60–63). β-glucan activate specific receptors or proteins, such as Dectin-1, toll-like receptors (TLR), complement receptor 3 (CR3), scavenger receptors (SR), and lactacyl ceramide (LacCer), triggering the secretion of cytokines, and subsequently activating other anti-tumor immune cells (64–66) (**Figure 3A**).

## 5 The role and biological mechanism of β-glucan in the prevention and treatment of digestive system neoplasms

It is well known that β-glucan, as an immune modulator, can effectively activate the immune system (67, 68). It exerts anti-tumor effects by activating innate and adaptive immune responses (69–71). Recent studies have shown that β-glucan has anti-tumor effects in colorectal cancer (54), pancreatic cancer (24) and gastric cancer (25). Furthermore, in animal models of gastric cancer, β-glucan can serve as a drug carrier to enhance the retention time of drugs at the tumor site, further augmenting the anti-tumor effects (17, 72). The biological mechanism of its role is still being explored (**Figure 3B**).

### 5.1 The therapeutic effect and mechanism of β-glucan on colorectal cancer

In animal models of colorectal cancer, Liu N et al. found that β-glucan can exert the following biological functions, including: 1) anti-inflammatory effect; 2) inhibition of cell proliferation; 3) promotion of cell apoptosis; 4) regulation of intestinal flora disorder; 5) improvement of intestinal barrier function; 6) increase of short-chain fatty acids (SCFA), which can exert anti-tumor effects through synergistic effects of the above biological functions (14). Qi J et al. found that alternating intake of β-glucan in the diet of mice with colon cancer can reduce colon damage and mortality by reducing TNF-α levels, increasing the relative abundance of Parabacteroides, and downregulating three genes associated with inflammation and cancer (*Hmgcs2*, *Fabp2*, and *Gpt*) *(* 73). IL-22 binding protein (IL-22BP) play an important role in inhibiting the development of intestinal tumors (74). In a mouse colorectal tumor model, low molecular weight β-glucan laminarin can exert its anti-colon tumor effect by regulating the Dectin-1-PGE2-IL-22BP signaling axis (75). Kim JH et al. found that low-molecular-weight *Aureobasidium pullulans-*fermented β-D-glucan (LMW-AP-FBG) can exert anti-tumor effects in mice with CT-26 colon cancer cells by reducing tumor proliferation, inducing apoptosis, and inhibiting inflammation (by inhibiting the NF-κB signaling pathway) (76). Kopiasz et al. found that low-molecular- weight β-glucan (derived from *oats*) can stimulate autophagy and increase apoptosis levels to positively affect early colorectal cancer rats (77). Sulaiman Binmama et al. found that in the mouse colon cancer model induced by azomethane (AOM) and injected subcutaneously with cancer cells, and found that *S.cerevisiae* isolated whole glucan particles (WGP) enhance the energy status of macrophages by activating Dectin-1, thereby inhibiting tumor growth (78). It is believed that *S.cerevisiae* or β-glucan can be used to prevent the occurrence of colon cancer. A recent study has elucidated the impact of a sustained 8-week dietary intake of low-molecular-weight oat β-glucan on the antioxidant capability, inflammatory markers, and colonic metabolomic profiles in a rat model of early colon cancer induced by azoxymethane (AOM). The findings reveal that β-glucan intervention significantly enhances total antioxidant status, diminishes superoxide dismutase (SOD) activity, and lowers thiobarbituric acid reactive substances (TBARS) levels. Notably, β-glucan supplementation was found to reduce proinflammatory cytokines including interleukin (IL-1α, IL-1β, IL-12 and C-reactive protein (CRP), while augmenting IL-10 concentrations. Moreover, metabolomic analyses have validated that oat β-glucan elevates the abundance of metabolites such as amino acids, purines, biotin, and folic acid. The researchers postulate that during the incipient stages of carcinogenesis, colonic metabolism undergoes profound alterations, and low-molecular-weight oat β-glucan emerges as a promising dietary supplement for modulating these mechanisms (79). Liu N and colleagues achieved precise fractionation of β-glucan isolated from lentils into three fractions with varying weight-average molecular weights (Mw) through ultrasonic degradation, and thoroughly evaluated their therapeutic potential in colorectal cancer. In cellular experiments, these β-glucan fractions exhibited remarkable inhibitory effects on colon cancer cell proliferation, induced apoptosis, and alleviated inflammatory responses. Moreover, *in vivo* investigations utilizing mouse models highlighted the superior anti-inflammatory and anti-colorectal cancer properties of low-molecular-weight β-glucan, which was attributed to its ability to restore the intestinal mucosal barrier, enhance the content of SCFAs, modulate the metabolic landscape of intestinal microbiota, and reshape the intestinal microbiota composition. These findings offer compelling scientific evidence for the utilization of β-glucan as a modulator of intestinal microbiota, representing a promising alternative therapeutic strategy for the clinical treatment of colon cancer (14). The impact of β-glucan on intestinal immune responses during colitis-associated colorectal cancer (CAC) remains unclear. Researchers have delved into the underlying mechanisms of β-glucan’s role in the pathogenesis of CAC. Following oral administration of β-glucan in a CAC mouse model, a significant increase in antitumor DCs within the tumor microenvironment was observed, leading to a subsequent augmentation of CD8+ T cells and associated cytokine production. Additionally, β-glucan enhanced resilience against chronic colitis by remodeling the inflammatory microenvironment. These data suggest that β-glucan can ameliorate experimental intestinal inflammation and delay the progression of CAC (80). Ji Hyeon Kim and colleagues evaluated the therapeutic potential of LMW-AP-FBGin BALB/c mice implanted with subcutaneous CT-26 colon cancer cells. Their study demonstrated that daily intraperitoneal injection of LMW-AP-FBG (5 mg/kg) over a two-week period significantly suppressed tumor growth by reducing tumor proliferation and inducing apoptosis. Moreover, the treatment reduced the viability of CT-26 cells in a dose-dependent manner, via mechanisms that involve the loss of mitochondrial transmembrane potential and the promotion of apoptosis. This investigation indicates that LMW-AP-FBG possesses anticancer properties both *in vitro* and *in vivo (* 76). The above studies suggest that β-glucan plays a certain preventive and therapeutic role in preclinical models of colorectal cancer, and further in-depth study of its biological mechanism of action in the future will lay the foundation for its clinical translation.

### 5.2 Therapeutic effect and mechanism of β-glucan on pancreatic cancer

Research indicates that β-glucan, as an immune agonist, has the ability to train the body’s immune system and promote anti-tumor immune effects by training and enhancing the body’s immune function (81, 82). In the mouse pancreatic cancer (PC) model, β-glucan can induce training immunity in mouse pancreas and prolong the survival rate of PC; the combination therapy with irreversible electroporation (IRE) showed better anti-tumor effects in a PC mouse model. The biological effects of this combination therapy mainly include: 1) Enhancing immune cell infiltration into the PC tumor microenvironment; 2) Enhancing the training response of tumor infiltrating bone marrow cells. 3) Reducing local and distant tumor burden and prolong the survival rate of mouse *in situ* PC model (24). Due to the highly desmoplastic tumor microenvironment (TME) of pancreatic ductal adenocarcinoma (PDAC), its treatment prognosis is poor. Researchers have developed an oral β-glucan-functionalized zinc doxorubicin nanoparticle system (β-Glus-ZnD-NPs) for targeted PDAC therapy. This β-Glus-ZnD NPs system actively targets and traverses the folds of the small intestine, overcoming the intestinal epithelial barrier. Subsequently, the nanoparticles are phagocytosed by endogenous macrophages (β-Glus-ZnD@Mϕ), which are activated to produce matrix metalloproteinases that destroy the desmoplastic stromal barrier. These macrophages also differentiate into an M1-like phenotype, modulating the tumor microenvironment and recruiting effector T cells, ultimately inducing tumor cell apoptosis (83). The liver is the most common site of metastasis in PDAC. A study indicates that β-glucan can activate liver-resident macrophages (Kupffer cells) and prevent PDAC from metastasizing to the liver. β-glucan exerts its anti-tumor metastasis effect through the following mechanisms: 1) upregulating the antigen presentation pathway in Kupffer cells; 2) driving the interaction between Kupffer cells and T cells; 3) stimulating T cells -mediated anti-tumor immunity; 4) Kupffer cells and T cells coordinate the activation of macrophages (BMDM). At the same time, researchers also found that the combination of β-glucan treatment and anti-PD1 treatment can inhibit liver metastasis. Geller AE and other studies have found that β-glucan enters the pancreas in a dectin-1-dependent manner, and then β-glucan stimulates trained myeloid cells to flow into the pancreas to exert anti-tumor effects (84). Researchers used CCR2^−/−^ mice to confirm that the peripheral immune cells that fight tumors are mainly trained CCR2^+^ myeloid cells, which recruit and infiltrate into pancreatic tumor cell areas through the CCL2-CCR2 signaling axis, and then exert their anti-tumor effects through phagocytosis and ROS-mediated cytotoxicity (84). To sum up, in the treatment of pancreatic cancer, a “cold tumor”, β-glucan primarily trains immune cells by acting as an immunostimulator, thereby mediating anti-tumor immune responses.

### 5.3 The therapeutic effect and mechanism of β-glucan on gastric cancer

Lentinan, a β-glucan extracted from mushrooms, has been proven to possess antitumor activity (25, 85). Researchers have confirmed its preventive and therapeutic effects on gastric cancer, and the mechanisms of its action include: 1) inhibiting the G2/M phase of the gastric cancer cell division cycle to exert anti-proliferative effects; 2) Lentinan can attenuate the wound healing, colony formation, and migration abilities of AGS cells; 3) Lentinan can increase the expression of phospho-p38, while reducing the expression of phospho-*ERK1/2* and *Mu-2*-related death-inducing gene (*MuD*) proteins; 4) Lentinan induces the generation of reactive oxygen species (ROS), directly participating in cell death (25). In the treatment of gastric cancer, β-glucan can be used as a main component of drug carriers to jointly exert anti-tumor effects. To develop an oral drug that improves treatment efficacy by prolonging gastric treatment time, researchers have developed an oral delivery tool based on β-glucan, which can control the release of *Bcl2* siRNA and 5-fluorouracil (*5FU*) payloads for more than 6 hours. By increasing their adsorption time in gastric mucus, *Bcl2* siRNA can selectively knock out the *Bcl2* gene to promote cell apoptosis and alleviate cancer (17). The team developed the therapeutic effect of oral administration of Navi/siRNA mediated by Glucan and Docosahexaenoic Acid (GADA) was studied in a mouse model of β-gastric cancer (72). The results showed that the mice treated with GADA/NAVI/siRNA had a higher inhibitory effect on *Bcl2*. In summary, in the treatment of gastric cancer, β-glucan not only plays a direct anti-tumor role, but also can be used as a major component of drug carriers to increase the adhesion properties of the carrier, thereby improving the duration and efficacy of drug treatment. These studies have opened up a new horizon for long-term oral drug therapy for gastric cancer.

## 6 Application of β-glucans in the treatment of patients with gastrointestinal tumors

For patients with advanced gastrointestinal cancer, it is highly desirable to continue chemotherapy with minimal adverse reactions for an extended period. Previous clinical studies have indicated that the adjunctive use of β-glucan during chemotherapy or radiotherapy for hepatocellular carcinoma, gastric cancer, and colorectal cancer can increase the 5-year survival rate by 15% and reduce the recurrence rate by 43% (26–28). In Japan, two types of β-glucans, krestin and lentinan, are licensed as drugs for gastric cancer treatment (85). Among them, Krestin is extracted from Coriolus versicolor, and this preparation has been clinically used for surgical treatment of resectable gastric cancer (86–88). Okuno K et al. investigated the effects of an oral β-glucan immunomodulator, Lentinula edodes mycelia extract (LEM), on immune function and chemotherapy-induced adverse reactions in 1 patient with gastric cancer and 7 patients with colorectal cancer. After treatment with LEM (1800 mg/day for 28 days), 8 patients experienced reduced adverse reactions such as nausea and abdominal pain caused by chemotherapy. Additionally, LEM increased the production of interferon (IFN)-γ by CD4+, CD8+ T, and CD56+ NK/NKT cells. These results suggest that the concurrent use of LEM with chemotherapy can reduce the probability of adverse reactions caused by cancer chemotherapy in patients with advanced cancer (89).

Lentinan (LNT) is an immune adjuvant medicine for advanced gastric cancer in Japan, in a multicenter clinical study conducted in Japan, 71 patients with advanced colorectal cancer who met the inclusion criteria were enrolled. In this study, researchers employed superfine dispersed lentinan (SDL), an oral formulation of 1,3-β-glucan, as an adjunctive therapy to assess post-chemotherapy adverse reactions, quality of life (QOL), and the binding capacity of peripheral blood monocytes (PBMs) to lentinan (LNT). After receiving SDL treatment (15 mg/day for 84 days), QOL was significantly improved in the majority of patients. Notably, the PBM binding rate to LNT was significantly higher in the QOL-improved group compared to the non-improved group. These results demonstrate that SDL is both safe and effective in suppressing chemotherapy-induced adverse reactions and enhancing QOL in patients with advanced colorectal cancer. Furthermore, the binding capacity of PBMs to LNT may serve as a potential predictor of QOL improvement following SDL administration (30). In a retrospective study examining the effects of oral β-glucan administration on chemotherapy-induced adverse reactions in 62 patients with advanced colorectal tumors, significant reductions in chemotherapy-related adverse reactions such as oral mucositis and diarrhea were observed following treatment with β-glucan (50 mg/day for 7 days). Notably, there were no significant decreases in white blood cell count, neutrophil count, or platelet count following chemotherapy. These findings suggest that β-glucan may be utilized to alleviate adverse reactions following chemotherapy in patients with advanced colorectal cancer (29) In patients with KRAS-mutant colorectal cancer (CRC), monotherapy with cetuximab has been ineffective. Researchers have explored the combination of β-glucan (Imprime PGG) with cetuximab, owing to Imprime PGG’s ability to enhance the recognition of natural immune cells. The study revealed that the combination of Imprime PGG and cetuximab demonstrated convincing yet moderate clinical activity, providing evidence for the clinical efficacy of Imprime PGG when combined with complement-activating antibodies (90). In a clinical study, β-glucan was incubated with the blood of colorectal cancer patients, and it was found that after incubation with β-glucan, the DNA damage parameters in the blood significantly decreased, suggesting that β-glucan can significantly reduce DNA damage in colorectal cancer patients through antioxidant effects (91). In a phase I clinical trial evaluating β-glucan-based combination therapy for PDAC, researchers aimed to assess the therapeutic efficacy of BTH1704 (a monoclonal antibody targeting aberrantly glycosylated Mucin 1) and Imprime PGG (a glucan derived from yeast that is crucial in triggering leukocyte-mediated cytotoxic responses against tumor cells), combined with gemcitabine, in patients with advanced PDAC (NCT02132403).

## 7 Summary and prospect

β-Glucan, as an immunomodulator, has demonstrated promising anti-tumor effects in preclinical studies of colorectal cancer, pancreatic cancer, and gastric cancer (14, 24, 25). Notably, the high stability of β-glucan renders it a crucial component for serving as a carrier for targeted drugs against gastric cancer (17). In clinical studies, β-glucan has been shown to improve the 5-year survival rate of hepatocellular carcinoma, gastric cancer, and colorectal cancer, while reducing the recurrence rate (26–28). Additionally, it can mitigate adverse reactions caused by chemotherapy, such as nausea, abdominal pain, mucositis, diarrhea, and leukopenia/thrombocytopenia (29), leading to an improvement in patient quality of life (30). Furthermore, β-glucan has the potential to enhance the anti-tumor effects of anti-tumor immune agents (90). Given that β-glucan is mostly derived from edible mushrooms, its natural origin promotes the development of β-glucan-related drugs or clinical health products. In the future, β-glucan may serve as a combined treatment method with monoclonal antibodies to improve anti-tumor immune responses. Although β-glucan has been proven to exert certain therapeutic effects in the treatment of gastrointestinal tumors, most of the studies demonstrating its anti-tumor effects are preclinical. More clinical research is needed to evaluate its therapeutic efficacy in gastrointestinal tumors. Simultaneously, further basic research is required to explore the specific biological mechanisms of β-glucan in the prevention and treatment of gastrointestinal tumors. In the future, β-glucan may serve as an adjuvant therapy for gastrointestinal tumors, benefiting patients with such malignancies.

## Author contributions

MW: Writing – original draft, Writing – review & editing. JP: Writing – original draft, Writing – review & editing. WX: Writing – review & editing. ZY: Writing – review & editing, Visualization. YZ: Writing – review & editing. JW: Writing – original draft, Writing – review & editing. AZ: Writing – review & editing.
